# Catastrophic health care expenditure and impoverishment in Bhutan

**DOI:** 10.1093/heapol/czac107

**Published:** 2022-12-07

**Authors:** Jayendra Sharma, Milena Pavlova, Wim Groot

**Affiliations:** Department of Health Services Research, CAPHRI, Maastricht University Medical Center, Faculty of Health, Medicine and Life Sciences, Maastricht University, PO Box 616, Maastricht 6200 MD, The Netherlands; Department of Health Services Research, CAPHRI, Maastricht University Medical Center, Faculty of Health, Medicine and Life Sciences, Maastricht University, PO Box 616, Maastricht 6200 MD, The Netherlands; Department of Health Services Research, CAPHRI, Maastricht University Medical Center, Faculty of Health, Medicine and Life Sciences, Maastricht University, PO Box 616, Maastricht 6200 MD, The Netherlands

**Keywords:** Bhutan, financial protection, catastrophic health expenditures, impoverishment, out-of-pocket payments

## Abstract

Monitoring financial hardship due to out-of-pocket spending on health care is a critical determinant of progress towards universal health coverage. This study investigates the occurrence, intensity and determinants of catastrophic health expenditure and impoverishment in Bhutan using three rounds of the cross-sectional Bhutan Living Standard Surveys carried out in 2007, 2012 and 2017. We use a composite financial hardship measure defined as households experiencing either catastrophic health expenditure or impoverished/further impoverished due to health spending or both. We calculated concentration indices to examine socio-economic inequalities. We used logistic regression to examine the factors associated with financial hardship. We find that, in the context of a significant increase in living standards, there is a sharp increase in the incidence of catastrophic health expenditure (using 40% of capacity to pay) and impoverishment (based on equivalized average food-share-based poverty line) between 2007 and 2017. In 2017, catastrophic health expenditure was estimated at 0.51%, impoverishment at 0.32% and further impoverishment at 1.93% of the population, cumulating to financial hardship affecting 2.55% of the population. Financial hardship particularly burdened rural dwellers and poorer households. Transportation costs almost doubled the risk of facing financial hardship. Households that were poor, had an unemployed head, were larger and had more elderly members had higher odds of financial hardship. This evidence should prompt policy and programmatic interventions to support Bhutan’s progress towards universal health coverage.

Key messages

- Despite an overwhelming state presence in health care and health services being provided free at the point of use, catastrophic health expenditure and impoverishment are prevalent in the Bhutanese society.
- The financial burden of health care has increased in the last decade and has particularly burdened rural dwellers, poorer households and certain vulnerable households.
- The health system needs to ensure better access to an appropriate level and quality of health services, including exploring demand-side financing for transport-related costs, to enhance the effective coverage of services and to reduce financial burden, especially in the rural communities and regions of the country and among the less advantaged population groups.

## Introduction

The United Nations General Assembly passed a political declaration on universal health coverage (UHC) in 2019, calling for countries to address high out-of-pocket (OOP) expenditure by providing programmes to ensure financial risk protection and eliminate impoverishment due to health-related spending, with a particular emphasis on the poor and vulnerable (United Nations, 2019). This is considered an important declaration since OOP payments are a reason for millions of people worldwide facing catastrophic health expenditure and economic impoverishment globally, reducing peoples’ ability to pay for other essential goods and services (Xu *et al.*, 2007; Wagstaff *et al.*, 2018a,b).

In essence, OOP expenditure on health consists of formal or informal payments, without the possibility of reimbursement, made by people while using any health care product or service. These expenditures are considered catastrophic if they exceed a pre-defined threshold of the household budget or capacity to pay, which could potentially lead to disruption to their living standards. At the same time, OOP expenditure is considered to be impoverishing when it pushes households below a pre-defined poverty line or further into poverty if they are already poor. The impoverishment measure has the added advantage of directly estimating the hardship caused by OOP expenditures. These two measures are crudely summarized as a measure of the risk and a measure of the impact, respectively. They have increasingly become the basis of empirical work in an increasing number of countries and formed the basis of global monitoring of Sustainable Development Goals (World Health Organization and World Bank, 2021).

Several global comparative studies (Xu *et al.*, 2003, 2007; van Doorslaer *et al.*, 2006, 2007; Wagstaff *et al.*, 2018a,b) have applied those measures in the last decades. Earlier studies show that ∼100 million people worldwide were forced into poverty, and ∼150 million people faced catastrophic expenditure while accessing health care each year (Xu *et al.*, 2007). More recent evidence featuring progress in 133 countries revealed that the global incidence of catastrophic health spending at the 10% threshold of household consumption has increased from 9.7% in 2000 to 11.4% in 2005 and to 11.7% in 2010 (Wagstaff *et al.*, 2018a). In 2010, 97 million people or 1.4% of the world population (at 1.90$ per day poverty line) and 122 million people or 1.8% of the world’s population (at 3.2$ per day) were impoverished by OOP spending on health, with Asia and Africa having the highest incidence of impoverishment (Wagstaff *et al*., 2018b).

There have also been efforts to consolidate the two indicators through a unified methodology (Wagstaff and Eozenou, 2014) and an integrated presentation of the two indicators to reflect aggregated financial hardship (Thomson *et al.*, 2016). Debates on the usefulness of these indicators for global comparative analysis have underscored the lack of a robust theoretical basis, reliance on poor quality survey data and several outstanding conceptual challenges underpinning the meaning of financial protection (Grépin *et al.*, 2020). As research interest in financial protection has grown, the methodology has evolved. Nevertheless, the two indicators assume a prominent place in measuring country and global progress towards UHC. Thus, they play an important role in health system monitoring and UHC policy dialogue, particularly in low- and middle-income countries.

In Bhutan, providing health care without financial hardship has been a continuing policy priority of the government and is considered a critical precondition to the national development paradigm of Gross National Happiness. The principle of Gross National Happiness underscores Bhutan’s development objective as a definitive improvement in the happiness and satisfaction of the people rather than a mere improvement in economic circumstances. All national policies, including health, go through the Gross National Happiness screening process. The health system, developed and modernized over the last six decades (Thinley *et al.*, 2017), is predominantly public, with the government taking charge of financing and delivery of health care services. This framework has resulted in significantly improved population health outcomes; Bhutan has been among the top global performers in life expectancy gains in the past 40 years (Thinley *et al.*, 2017). Health services are delivered through three levels of hierarchical structure with outreach clinics, sub-posts and primary health centres at the primary level, district hospitals at the secondary level and national and regional referral hospitals at the tertiary level. The provision of public services is mostly free of charge at the point of use, including referral abroad for treatment unavailable in the country. There are exclusions to this free-of-charge package, namely private cabins and off-hour consultations or clinical services in government hospitals, cosmetic surgical and selected dental care and cost for obtaining medical certificates. While private sector participation in health care has historically been limited to retail pharmacy shops, there has been a gradual increase in their presence in the last decade. In 2014, the government opened diagnostic services to private sector participation, and ∼14 private diagnostic centres currently operate, primarily in the four major urban centres in the country (Thinley *et al.*, 2017). Recently, there has been an increase in the number of Bhutanese citizens accessing health care abroad of their own choice, particularly for medical screening, pregnancy and delivery care. While the magnitude of these expenditures has not been assessed, anecdotal evidence raises important questions relating to the amount of OOP payments and the associated financial burden on households.

The national health accounts published by the government reported health expenditure as a share of Gross Domestic Product at 4% and 4.5% in the financial year 2018–19 and 2019–20, respectively (Ministry of Health, 2021). The government share of the current health expenditures was 70.2% and 73.4%, and households’ contribution (OOP payments) to the current health expenditures were 18% and 15.4%, respectively, while the rest were financed by development assistance, enterprises and voluntary insurance, in these 2 years (Ministry of Health, 2021).

Thus, the policy of free health care, while an important enabler, cannot be considered a panacea for financial protection in health care. The population could be availing care on their own, foregoing care or availing alternative care due to choice or service delivery constraints. A previous study using data from 2012 estimated the incidence of catastrophic payment in Bhutan at 4.06% at 10% of household budget threshold and 1.45% at 25% of household budget threshold (Wang *et al.*, 2018). The same study reported that slightly >2000 people at a 1.9$ international poverty line and 7000 people at a 3.2$ international poverty line were pushed below the poverty line due to OOP expenditure for health care (Wang *et al.*, 2018). There is a continuing need to closely monitor financial protection in health care as part of Bhutan’s health policy and UHC objectives.

This study presents a detailed analysis of catastrophic health care expenditure and impoverishment using three rounds of the Bhutan Living Standard Survey (BLSS) covering a time span of 15 years. The objectives of this study are to (1) estimate the incidence, intensity and distributional profile of catastrophic health expenditures and impoverishment and (2) examine the factors that are associated with financial burden as a result of OOP expenditure in Bhutan. These analyses are expected to contribute to the UHC policy dialogue in Bhutan as well as contribute to the international discourse on catastrophic health expenditure and impoverishment.

## Methods

### Data

We used the secondary data from the ‘Public User File (PUF)’ of 2007, 2012 and 2017 rounds of the BLSS. Four rounds of the survey have been completed to date. The 2003 round has been excluded from this analysis in view of inconsistencies in the sampling frame and limitations in data on the variables of interest. The BLSS 2007, 2012 and 2013 rounds are nationally representative surveys designed to cover all 20 districts, both urban and rural areas, and use a stratified two-stage sampling design. The sample sizes across these rounds were 9798, 8968 and 11 660 households, respectively. Samples were drawn from the preceding Population and Housing Censuses. Institutional households were excluded from the sampling frame across all rounds of the survey. Details about the sampling procedure can be found in the BLSS 2017 report (National Statistical Bureau, 2017). Trained enumerators visited the sampled households and collected data, with three failed attempts to make contact with household members counted as non-response.

The BLSS questionnaire covers various aspects of living standards and social well-being, including demography, health, education, housing, household income and expenditures and the use of and satisfaction with public services. Information on OOP expenditure is derived from the health module questionnaires, which go into much more detail in every subsequent wave. The BLSS 2007 asked the total expenditure on health care, irrespective of whether it was availed within or outside the country, categorized into consultation fees, medicines and health accessories, transportation and other health expenditures, in the preceding 4 weeks. Since the 2012 wave, the health module covered cases of sickness or injury in the last 4 weeks and admission for an overnight stay at a medical facility in the last 12 months, with additional information on expenditure related to childbirth and rimdo/puja (religious ceremony). Expenditures on hospital stay, which were collected in a 12-month time frame, were converted into monthly averages. We classified health expenditure as per the ‘Classification of Individual Consumption According to Purpose’ (COICOP) revised guidance (United Nations, 2018) as much as the data would allow, which resulted in the removal of expenditures related to transport and rimdo/puja (religious ceremonies). However, considering the significance of the transport variable in the expenditure basket and the potential health policy implications, we performed additional analyses where transport costs were included in the OOP expenses with estimates for all critical indicators reported with and without transport costs. Details about the updates in the questionnaires used during these surveys are available in the BLSS 2017 report (National Statistical Bureau, 2017).

### Analysis of catastrophic health care expenditure

OOP expenditures on health services are considered catastrophic if they exceed a certain fraction of the total household resources (Wagstaff *et al.*, 2003; O’Donnell *et al.*, 2007) or capacity to pay (Xu *et al.*, 2003, 2007). Considering the limitation that using total household consumption or income could underestimate financial hardship among poorer households (Xu *et al.*, 2003; O’Donnell *et al.*, 2007, Thomson *et al.*, 2016), we chose the capacity-to-pay approach. The capacity-to-pay approach assumes that households have to meet basic needs before they can spend on health and considers the effective income remaining after basic subsistence needs have been met. Therefore, this approach represents the true capacity to pay for health care expenditure, derived after subtracting the subsistence need from the total consumption expenditure (Xu *et al.*, 2003, 2007). To account for economies of scale in household consumption, we used the Organization for Economic Co-operation and Development equivalence scale since Bhutan does not have a country-specific equivalence scale. This scale assigns a weight of 1 for the household head, 0.7 for additional adult members and 0.5 for each child.

To estimate subsistence needs, researchers have used several methods: actual food spending method (World Health Organization, 2000), food-share-based poverty line adjusted for household size (Xu, 2005; Xu *et al.*, 2007) and, more recently, the use of normative spending on food, housing and utilities method using not just food but household expenses on housing and utilities purported to be more relevant for middle- and higher-income countries (Thomson *et al*., 2016). We adopted the food-share-based poverty line for estimating household subsistence, the closest proxy of spending on basic needs in Bhutan, where we estimated the average food expenditures of households whose food expenditure share of the total household expenditure is within the 45th and 55th percentiles of the total sample. We reclassified food expenditure in the survey data to uniformly exclude alcohol and tobacco from the food consumption basket.

For households whose total expenditure is above the estimated subsistence need, capacity to pay is calculated as the total consumption expenditure minus the estimated subsistence needs. For households whose total expenditure is below the estimated subsistence need, capacity to pay is taken as total consumption expenditure minus food spending. Consistent with the predominant literature on this approach (Xu *et al.*, 2003, Xu, 2005), a household is considered to have incurred catastrophic payments if OOP expenditure exceeds 40% of the capacity to pay.

To measure the intensity of catastrophic health expenditure, and as complementary statistics, we calculated the ‘overshoot’ of catastrophic health expenditure, which represents the average extent to which health care expenditure exceeds the respective threshold (in this study, 40% of the capacity to pay), and the ‘mean positive overshoot’, which represents only those households incurring OOP payments (O’Donnell *et al.*, 2007).

### Analysis of impoverishment due to OOP health care expenditure

OOP expenditure is considered to be impoverishing when it pushes households below a pre-defined poverty line or further into poverty if they are already poor according to that poverty line. As discussed earlier, we adopted a food-share-based poverty line equivalized for household size as a measure of household subsistence. Building on Wagstaff and Eozenou (2014) and Thomson *et al.* (2016), we summarized the impoverishment impact on the population by categorizing the population into five mutually exclusive groups in relation to the equivalized food-share-based poverty line: (1) population with no OOP spending; (2) population with OOP spending but with no risk of impoverishment; (3) population at risk of impoverishment, i.e. those 20% falling nearest to the poverty line after OOP expenditure; (4) population who are impoverished after OOP spending and (5) population who are further impoverished, i.e. those who were below the poverty line even before the OOP expenditure. An additional measure of changes in the poverty gap has been employed to estimate the impact of OOP health expenditure on poverty. The poverty gap is equal to the fraction of the population who are poor multiplied by the average income shortfall below the selected poverty line (O’Donnell *et al.*, 2007). In other words, it measures the depth to which individuals have fallen below the poverty line, providing an estimate of the financial resources necessary to raise that individual to the poverty line. To estimate the changes in the poverty gap, we measured the differences in poverty gaps using household total consumption gross and net of household OOP spending, estimated in national currency units.

### Estimation of composite financial hardship due to OOP health care expenditure

Catastrophic expenditure and impoverishment as a result of OOP expenditure for health care are calculated separately and often seen as two different measures, indicating different aspects of financial hardship. However, the measures of catastrophic expenditure and impoverishment are not mutually exclusive. Building on previous work by Wagstaff and Eozenou (2014) and Thomson *et al.* (2016), we estimate the composite financial burden on a household by combining the measures of catastrophic health expenditure and impoverishment. We define composite financial hardship as the population experiencing either catastrophic health expenditure or impoverished/further impoverished due to health spending or both. Consequently, for a population to qualify as facing financial hardship, at least one of the following criteria should apply: (1) they incurred catastrophic health expenditure, (2) they were impoverished after OOP spending or (3) they were already below the poverty line and further impoverished by OOP spending.

### Socio-economic inequality in catastrophic payments, impoverishment and financial hardship

We calculated concentration indices to estimate socio-economic inequality in catastrophic health expenditure, impoverishment and composite financial hardship faced by households in the different rounds of the survey. Concentration indices are derived from the concentration curves, which plot the cumulative percentage of the health variable against the cumulative percentage of the population ranked by the living standards measure. It is measured as twice the area between the curve and the 45° line, i.e. the line of equality (O’Donnell *et al.*, 2007). Considering that the outcome measures are binary bounded variables, we chose to compute the corrected Erreygers concentration index (Erreygers, 2009; Kjellsson and Gerdtham, 2013). A positive value of the index indicates that the richest households have a greater tendency to incur catastrophic health expenditure, impoverishment or financial hardship, and a negative value shows a greater tendency among the poorest.

### Factors associated with financial hardship

We pooled the data for all three rounds of the survey and applied logistic regression to examine the covariates of financial hardship incurred by households. We estimated two multivariate models; model 1 was estimated by accounting for OOP expenditure alone, and model 2 was estimated with transportation cost added to OOP expenditure as discussed earlier. We selected a set of covariates containing information about demographic and socio-economic status, which found relevant in previous studies. The covariates and the variable types specified in the analysis were household head’s age (continuous), gender (binary, male/female), education (categorical, no education/primary, secondary or vocational/bachelors and higher) and employment status (binary, employed/unemployed), household socio-economic status (categorical, quintile 1–5), household size (continuous), the number of children <13 years old (continuous), the number of elderly persons in the household (continuous) and geographical region (categorical, western region/central region/eastern region) and area (binary, rural/urban). The dependent variable was constructed as a dichotomous variable where 0 and 1 denote the households experiencing or not experiencing financial hardship. All analyses were performed in Stata (version 13.1).

## Results

### Household consumption and OOP health care expenditure

Table 1 provides a summary of household consumption and OOP expenditure across the survey periods. In nominal terms, there is a significant increase in household food and total consumption expenditures, with the estimated relative food-share-based poverty line close to doubling across the subsequent survey period/5 years. The increase in OOP expenditure does not follow a similar trend, registering a more moderate increase over the survey periods in nominal terms and registering a slight decline in the latest survey round (2017). The mean monthly household OOP payment is Ngultrum 125.35 (1.9 USD^1^), Ngultrum 361.18 (5.4 USD) and Ngultrum 344.02 (5.2 USD) in 2007, 2012 and 2017, respectively. At 2019 prices, the mean household monthly OOP payment was Ngultrum 467.98 (7.0 USD), Ngultrum 871.03 (13.1 USD) and Ngultrum 570.83 (8.6 USD), respectively, in 2007, 2012 and 2017. Medicine cost dominates the OOP basket across all the survey periods. Transportation cost for accessing health care is a significant indirect cost item registering a third to a half of the total cost of accessing health care. The mean household OOP expenditures are consistently and proportionately concentrated among the richer households. The results have been derived from a sample of 9798 households in 2007, 8968 households in 2012 and 11 660 households in 2017. The socio-demographic profile of the respective samples is elaborated in Supplementary Appendix 1.

**Table 1.** Distribution of household consumption and health care expenditure

	2007 *n* = 9798	2012 *n* = 8968	2017 *n* = 11 660
Mean monthly household total consumption expenditure, in Ngultrum^a^	13 822.59	18 367.32	33 648.11
Mean monthly household food expenditure, in Ngultrum^a^ (as percentage of monthly household total consumption expenditure)	5110.30 (36.97%)	6857.54 (37.34%)	14 120.37 (41.96%)
Mean monthly household subsistence expenditure, in Ngultrum^a^	5866.20	8131.12	15 092.56
Food-based relative poverty line, in Ngultrum^a^	1407.92	2127.82	4194.89
Mean monthly household OOP expenditure—not including transport costs, in Ngultrum (as percentage of monthly household total consumption expenditure)	125.35 (0.91%)	361.18 (1.97%)	344.02 (1.02%)
Additional monthly costs related to transportation to access health care, in Ngultrum^a^	98.00	259.35	198.49
Mean monthly household health expenditure, including transport costs, in Ngultrum^a^ (as percentage of monthly household total consumption expenditure)	223.35 (1.62%)	620.53 (3.38%)	542.51 (1.61%)
Mean monthly household expenditure on different components of OOP, including transport cost, in Ngultrum^a^ (as percentage of mean monthly household OOP expenditure)	Medicine 74.51 (33.36%)	Medicine 211.34 (34.06%)	Medicine 218.23 (40.23%)
	Medical consultation 23.22 (10.40%)	Outpatient 125.31 (20.19%)	Outpatient 109.03 (20.10%)
	Health care–associated costs 27.62 (12.37%)	Inpatient 24.53 (3.95%)	Inpatient 16.76 (3.09%)
	Transport 98.00 (43.88%)	Transport 259.35 (41.79%)	Transport 198.49 (36.59%)
Mean monthly OOP expenditure, including transport cost, by consumption quintiles, in Ngultrum^a^			
Quintile 1 (poorest)	26.68	66.41	189.67
Quintile 2	64.31	170.52	400.73
Quintile 3	147.69	292.86	345.79
Quintile 4	180.02	591.36	504.64
Quintile 5 (richest)	698.28	1982.40	1272.47

Note: Weighted estimates.

Source: BLSS.

a Nominal terms in Bhutanese Ngultrum. 1 USD = 66.43 Ngultrum, based on the annual average for 2017 calculated by the Royal Monetary Authority of Bhutan

### Household representation and budget share

Table 2 illustrates the distribution and characteristics of households incurring OOP payments and the household budget share of OOP payments among households reporting these expenditures. Overall, there has been an increase in the proportion of households reporting OOP expenditure over the survey period with consistent urban dominance. Richer households more often have OOP payments, which is consistent over the survey periods. There is a wide variation between the districts.

**Table 2.** Household representation and budget share

	Proportion of households incurring OOP payments for health care (%)			OOP payments as a proportion of the total household expenditure for those households reporting OOP payments (%)		
	**2007** ***n* = 9798**	**2012** ***n* = 8968**	**2017** ***n* = 11 660**	**2007** ***n* = 9798**	**2012** ***n* = 8968**	**2017** ***n* = 11 660**
National estimate	17.60	29.18	25.06	2.99	4.10	3.02
Area of residence						
Rural	14.16	26.92	18.44	3.83	4.70	3.90
Urban	25.58	33.58	37.06	1.90	3.19	2.22
Geographical region						
Western region	25.20	32.53	33.26	2.63	3.58	2.60
Central western region	21.13	36.54	24.91	2.89	4.34	2.38
Central eastern region	9.70	21.91	19.62	3.07	3.96	3.63
Eastern region	8.29	23.40	14.47	4.68	5.04	4.88
Socio-economic status						
Quintile 1 (poorest)	7.20	14.62	11.11	3.39	3.60	4.61
Quintile 2	10.31	21.79	18.35	2.99	3.23	3.60
Quintile 3	15.36	26.86	22.86	3.54	3.60	2.58
Quintile 4	21.98	35.47	32.28	2.30	4.25	2.78
Quintile 5 (richest)	33.16	47.19	40.71	3.10	4.84	2.75
Administrative districts						
District (highest %)	34.01	44.73	43.36	6.24	10.63	10.60
District (lowest %)	3.03	9.76	4.78	0.85	1.52	1.71

Note: Weighted estimates.

Source: BLSS.

Table 2 also illustrates the household budget share of OOP payments among households reporting OOP expenditures. Overall, the composition of OOP in the household budget among those who have any OOP expenditure remained <5%. Rural households spent a larger proportion of their annual budgets on health care compared with urban households, and the region in the east, in general, registered a consistently high proportion than the rest of the country although there has been a wide difference among the districts.

### Catastrophic health care expenditure

The incidence and intensity of catastrophic health payments over the three rounds of survey periods are shown in Table 3. While we may require a longer data frame to appropriately gauge the trend, there is a sharp increase in the incidence of catastrophic health expenditure between 2007 and 2012. An estimated 3534 people faced catastrophic health expenditures in 2017 compared with 1763 people in 2007.

**Table 3.** Incidence and intensity of catastrophic expenditure

	2007 *n* = 9798		2012 *n* = 8968		2017 *n* = 11 660	
	OOP payments for health care	OOP payments for health care + transport cost	OOP payments for health care	OOP payments for health care + transport cost	OOP payments for health care	OOP payments for health care + transport cost
Headcount						
National	0.28 (0.06)	1.02 (0.12)	0.82 (0.18)	1.74 (0.18)	0.51 (0.09)	1.51 (0.16)
Rural	0.35 (0.08)	1.26 (0.15)	0.94 (0.17)	2.14 (0.25)	0.56 (0.12)	1.93 (0.22)
Urban	0.09 (0.06)	0.34 (0.11)	0.58 (0.13)	0.85 (0.15)	0.41 (0.12)	0.68 (0.16)
Quintile 1 (poorest)	0.07 (0.04)	0.82 (0.25)	0.21 (0.11)	0.49 (0.18)	0.37 (0.17)	2.20 (0.42)
Quintile 2	0.18 (0.10)	0.68 (0.19)	0.18 (0.11)	0.62 (0.22)	0.41 (0.15)	2.01 (0.42)
Quintile 3	0.34 (0.13)	1.23 (0.27)	0.79 (0.30)	1.43 (0.37)	0.44 (0.20)	1.42 (0.36)
Quintile 4	0.09 (0.07)	0.74 (0.22)	1.18 (0.35)	2.47 (0.47)	0.69 (0.26)	1.08 (0.31)
Quintile 5 (richest)	0.66 (0.22)	1.51 (0.32)	1.55 (0.33)	3.22 (0.51)	0.57 (0.18)	1.08 (0.28)
Overshoot	0.06 (0.02)	0.24 (0.04)	0.11 (0.04)	0.29 (0.04)	0.07 (0.02)	0.80 (0.18)
Mean positive overshoot	21.93 (3.45)	23.35 (2.06)	13.81 (1.25)	16.93 (1.25)	14.79 (2.01)	53.04 (10.26)

Notes: Weighted estimates. The values in parentheses are standard errors.

Source: BLSS.

In general, catastrophic health expenditure is slightly higher for the richer income groups, with rural areas consistently registering a higher incidence of catastrophic health expenditure. Transport cost has the potential to more than double the catastrophic expenditure incidence. The impact of transport on catastrophic health expenditure incidence is particularly pronounced in rural areas. On average, households spent 0.06–0.11% beyond the catastrophic threshold, without accounting for transport costs, in the various survey periods. The mean positive overshoot, which accounts for only those households who have incurred OOP expenditures, ranged from 13.81% to 21.93% in the different survey periods.

### Impoverishment due to OOP health care expenditure

**Figure 1.**
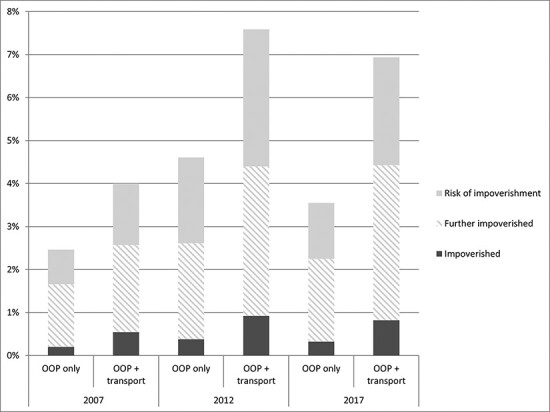
Impoverishment risk and magnitude

Table 4 summarizes the population risk and incidence of impoverishment in the respective survey periods. Overall, impoverishment peaked in 2012 when both the risk and magnitude of impoverishment were the highest (see Figure 1). Across all the survey periods, the proportion of the population already poor and incurring OOP payments had the highest share of impoverishment. If we extrapolate our results for 2017 with a total estimated population of 692 895, we find that 0.32% of the population or 2217 people were impoverished, 1.93% of the population or 13 372 poor people were further pushed into poverty and a further 1.3% or 9008 people were at risk of impoverishment, i.e. they stayed close to the poverty line. Transport costs almost doubled the magnitude of impoverishment, as well as substantially increased the poverty gap as a result of impoverishment.

**Table 4.** Impoverishment risk and magnitude

Proportion of population impoverished and at risk of impoverishment	2007 *n* = 9798	2012 *n* = 8968	2017 *n* = 11 660
OOP payments for health care			
No OOP	80.91%	68.79%	73.61%
OOPs but not at risk of impoverishment	16.63%	26.60%	22.84%
Risk of impoverishment	0.80%	2.00%	1.30%
Impoverished	0.20%	0.38%	0.32%
Further impoverished	1.46%	2.23%	1.93%
OOP payments for health care + transport cost			
No OOP	74.88%	58.84%	64.76%
OOPs but not at risk of impoverishment	21.12%	33.57%	28.30%
Risk of impoverishment	1.43%	3.19%	2.51%
Impoverished	0.54%	0.92%	0.82%
Further impoverished	2.03%	3.48%	3.61%
Increase in the poverty gap as a result of impoverishment (Ngultrum, nominal)	2007	2012	2017
Changes in the poverty gap due to OOP health expenditure (SE)	*0.89 (0.39)*	*1.51 (0.45)*	*3.92 (1.14)*
Changes in the poverty gap due to OOP health expenditure and transport cost (SE)	*2.57 (0.91)*	*3.87 (0.97)*	*25.65 (11.18)*

Notes: Weighted estimates; SE = standard error.

Source: BLSS.

### Composite financial hardship due to OOP health care expenditure

Table 5 presents the details of the population experiencing financial burden owing to OOP payments for health care, either facing catastrophic payments or impoverishment or both. The share of the population facing financial hardship increased from 1.87% in 2007 to 2.55% in 2017, with a steep increase in 2012. When transportation costs are included, the risk of facing financial hardship almost doubled. A major brunt of financial hardship is encountered in rural areas of the country, and while factoring in transport costs, the differences in financial hardship among rural and urban dwellers are more pronounced. Overall, 7.8% of the people in the poorest quintile faced financial hardship compared with 0.57% of the richest quintile in 2017.

**Table 5.** Proportion of population experiencing financial burden owing to OOP payments for health-care (either facing catastrophic payments or impoverishment or both), % (CI)

	2007 *n* = 9798		2012 *n* = 8968		2017 *n* = 11 660	
	OOP payments for health care	OOP payments for health care + transport cost	OOP payments for health care	OOP payments for health care + transport cost	OOP payments for health care	OOP payments for health care + transport cost
National	1.87 (0.77–2.96)	3.19 (1.38–5.00)	3.31 (1.58–5.05)	5.80 (3.32–8.28)	2.55 (1.17–3.92)	4.99 (2.51–7.48)
Rural	2.50 (2.01–3.00)	4.21 (3.59–4.82)	4.37 (3.62–5.11)	7.80 (6.82–8.79)	3.29 (2.70–3.89)	6.80 (5.99–7.61)
Urban	0.09 (−0.02 to 0.20)	0.35 (0.11–0.59)	0.97 (0.62–1.32)	1.34 (0.94–1.74)	1.07 (0.70–1.44)	1.39 (0.96–1.82)
Quintile 1 (poorest)	4.83 (3.57–6.09)	6.52 (5.11–7.92)	7.89 (6.12–9.66)	12.13 (9.94–14.32)	7.81 (6.11–9.51)	14.82 (12.69–16.96)
Quintile 2	2.35 (1.41–3.28)	4.01 (2.85–4.97)	4.62 (3.13–6.11)	7.84 (5.97–9.72)	3.96 (2.74–5.17)	8.34 (6.67–10.01)
Quintile 3	1.78 (0.88–2.67)	3.76 (2.55–4.97)	2.15 (1.06–3.24)	3.71 (2.33–5.08)	1.22 (0.50–1.95)	2.52 (1.51–3.52)
Quintile 4	0.45 (−0.11 to 1.02)	1.01 (0.34–1.67)	1.55 (0.71–2.39)	3.74 (2.39–5.09)	0.78 (0.24–1.32)	1.28 (0.62–1.94)
Quintile 5 (richest)	0.66 (0.22–1.10)	1.51 (0.88–2.15)	1.55 (0.89–2.20)	3.22 (2.21–4.22)	0.57 (0.22–0.92)	1.08 (0.53–1.63)

Note: CI = confidence interval.

Source: BLSS.

### Socio-economic inequality in catastrophic payments, impoverishment and financial hardship

Table 6 shows the concentration indices for catastrophic health expenditure, risk and incidence of impoverishment and overall financial hardship as a result of OOP expenditure for health care. We do not find significant evidence to suggest that catastrophic expenditure is concentrated among the poor population. However, all other indices related to impoverishment and financial hardship are negative, confirming that the financial burden of OOP payments is more concentrated amongst the poorer segment of the population. Concentration curves for the different rounds of the survey, with and without adding transport costs, are available in Supplementary Appendix 2.

**Table 6.** Concentration indices

	2007 *n* = 9798		2012 *n* = 8968		2017 *n* = 11 660	
	Index value	*P* value	Index value	*P* value	Index value	*P* value
OOP payments for health care						
Catastrophic	0.0035407	0.030	0.01186589	0.000	0.00210578	0.246
Impoverished	−0.00029353	0.808	−0.00464592	0.006	−0.00402883	0.002
Further impoverished	−0.03332313	0.000	−0.05329284	0.000	−0.05141154	0.000
Risk of impoverishment	−0.01166352	0.000	−0.02438998	0.000	−0.02191147	0.000
Composite financial hardship	−0.03056962	0.000	−0.04656503	0.000	−0.05118277	0.000
OOP payments for health care + transport cost						
Catastrophic	0.00486215	0.084	0.02353621	0.000	−0.00973481	0.005
Impoverished	−0.00170321	0.359	−0.00733414	0.015	−0.01050742	0.000
Further impoverished	−0.04438627	0.000	−0.07977834	0.000	−0.09641978	0.000
Risk of impoverishment	−0.01884846	0.000	−0.04190174	0.000	−0.03556431	0.000
Composite financial hardship	−0.039651	0.000	−0.06492125	0.000	−0.10103108	0.000

Source: BLSS.

### Factors associated with financial hardship

The results of multivariate logistic regression are summarized in Table 7. Both models estimated that the risk of financial burden is higher among poorer households, households with unemployed heads and those with more elderly members. Households in the richer quintiles are progressively less likely than the poorest quintile to run into financial hardship. In contrast, households with unemployed heads are ∼1.5 times more likely to incur financial hardship. With every additional elderly member in the household, the household is 20% more likely to experience financial hardship. The odds of households incurring financial hardship was 2.73 times in 2017 compared with 2007. Households with female heads and more children are less likely to incur financial hardship. When we consider transport costs together with other OOP payments, the risk of financial hardship in rural areas is 1.87 times that of urban areas.

**Table 7.** Results of logistic regression: factors associated with experiencing financial hardship

	Model 1 (OOP payments for health care)		Model 2 (OOP payments for health care + transport cost)	
	OR (CI)	*P* value	OR (CI)	*P* value
Urban	1		1	
Rural	1.30 (0.97–1.74)	0.084	1.87 (1.48–2.36)	0.000
Western region	1		1	
Central region	0.78 (0.62–0.98)	0.030	0.96 (0.80–1.14)	0.613
Eastern region	0.69 (0.54–0.87)	0.002	1.06 (0.89–1.26)	0.516
Male household head	1		1	
Female household head	0.71 (0.57–0.89)	0.003	0.75 (0.64–0.88)	0.001
Age of household head	1.01 (1.00–1.02)	0.026	1.01 (1.00–1.01)	0.010
Number of household members	1.50 (1.42–1.58)	0.000	1.45 (1.39–1.52)	0.000
Number of children <13 years of age	0.89 (0.81–0.97)	0.011	0.89 (0.82–0.95)	0.001
Number of elderly (>65) household members	1.22 (1.07–1.39)	0.003	1.23 (1.12–1.36)	0.000
Quintile 1 (poorest)	1		1	
Quintile 2	0.36 (0.28–0.45)	0.000	0.43 (0.36–0.51)	0.000
Quintile 3	0.14 (0.10–0.19)	0.000	0.19 (0.15–0.23)	0.000
Quintile 4	0.07 (0.05–0.11)	0.000	0.12 (0.10–0.16)	0.000
Quintile 5 (richest)	0.11 (0.08–0.16)	0.000	0.17 (0.13–0.22)	0.000
No formal education	1		1	
Primary, secondary and vocational	1.18 (0.91–1.52)	0.208	0.98 (0.81–1.20)	0.863
Bachelors and higher	0.47 (0.17–1.30)	0.144	0.42 (0.20–0.92)	0.029
Employed	1		1	
Unemployed	1.49 (1.19–1.85)	0.000	1.45 (1.23–1.70)	0.000
Year 2007	1		1	
Year 2012	2.42 (1.89–3.10)	0.000	2.38 (1.98–2.87)	0.000
Year 2017	2.73 (2.11–3.52)	0.000	2.96 (2.45–3.56)	0.000
Number of observations	30 405		30 405	
Pseudo *R*^2^	0.1704		0.1708	

Notes: OR = odds ratio; CI = confidence interval.

Source: BLSS.

## Discussion

Despite an overwhelming state presence in health care and public health services being provided free at the point of use, we find that a quarter of Bhutanese households spend as much as 4% of their household budget on health care, predominantly to purchase medicines. These findings must alert health policy circles which may ignore such issues considering the heavily state-provided/subsidized health care system. While richer households and richer geographical regions are spending more on OOP payments, the impact on the household budget is larger for poorer households and rural areas.

The interpretation of the risk and magnitude of OOP payments on households needs to be done with several qualifications considering the methodological diversity and choices of thresholds. Given the paucity of literature on Bhutan on this subject, direct comparisons of our findings are limited. However, considering that the global estimates of catastrophic health expenditure for Asia were 12.8% at the 10% of household budget threshold and 3.1% at 25% threshold in 2010 (Wagstaff *et al.*, 2018a) and impoverishment at 1.9% at 1.9$ international poverty line and 2.4% at 3.2$ international poverty line (Wagstaff *et al*., 2018b), the estimate for Bhutan is substantially below the Asian average. Among the World Health Organization Southeast Asian countries, Bhutan recorded the lowest catastrophic payment incidence after Thailand and Timor-Leste at 10% of the household budget threshold and the lowest rates of impoverishment after Thailand and Sri Lanka at 1.9$ international poverty line (Wang *et al.*, 2018). This reflects the strength of the current health system framework, which has the strong commitment towards strengthening primary health care for UHC (Ministry of Health, 2010) inspired by the national development philosophy of Gross National Happiness (Tobgay *et al.*, 2011).

Despite this relatively favourable status, our analysis shows that catastrophic payments are prevalent in Bhutan, have increased in the last decade and have particularly burdened the rural dwellers and poorer households. Trends in impoverishment were similarly contributing to the sharp increase in financial hardship from 2007 to 2012 and then staying more or less at that level thereafter. Households were almost three times more likely to incur financial hardship in 2017 compared with 2007. This steep rise from 2007 to 2012 is open to further investigation, and our data frame is inadequate to evaluate a long-term trend. However, improved living standards (National Statistics Bureau, 2017), increasing health-seeking behaviour and an increasing number of affluent Bhutanese availing health services abroad (Thinley *et al*., 2017) could have contributed to the increase in financial hardship in accessing health care over the years. Since the mid-2000, there have been incremental increases in private sector financing and provision of health care. These include the introduction of private health insurance, off-hour consultation at major public hospitals and establishment of private diagnostic centres in urban centres. These reforms may have mildly altered patterns of private spending on health care.

We demonstrate in this analysis that the financial burden on health care falls disproportionately heavier on the poor section of society and rural areas of the country. Our analyses also offer some insights into who amongst the Bhutanese population is most vulnerable to financial hardship as a result of OOP spending on health care. Households with an unemployed head, larger size and more elderly members have increased odds of financial burden. Female-headed households were at lower risk, conflicting evidence in several countries (Li *et al.*, 2012; Khan *et al.*, 2018; Pandey *et al.*, 2018; Myint *et al.*, 2019), and perhaps explained by the majority of traditionally matriarchal Bhutanese households. We see several consistencies in these findings with evidence on catastrophic health expenditure from neighbouring Asian countries. The unemployed household head was found to increase the odds of a household incurring catastrophic expenditure in China (Li *et al.*, 2012). Larger household sizes were found to increase the odds of incurring catastrophic expenditure in Myanmar (Myint *et al.*, 2019). Households having more elderly members increased the odds of catastrophic expenditure in India (Pandey *et al.*, 2018), China (Li *et al.*, 2012) and Nepal (Ghimire *et al.*, 2018). Similarly, the remote geographical factors played a role in increasing the odds of catastrophic expenditure in Bangladesh (Khan *et al.*, 2018), India (Pandey *et al.*, 2018), China (Li *et al.*, 2012), Nepal (Ghimire *et al.*, 2018) and Myanmar (Myint *et al.*, 2019).

We also find cross-regional differences. While households in the eastern region of Bhutan spent a higher proportion of their household budget on health, the overall risk of financial hardship was higher in the western part of the country, which mostly includes districts generally characterized by better socio-economic and poverty status (National Statistics Bureau, 2017). This raises important questions related to foregone care in the eastern region and pockets of high-risk groups in the western region. The concentration index of the overall financial burden is pro-rich, suggesting that the financial burden of OOP payments is more concentrated amongst the poorer segment of the population, while the pro-poor incidence of catastrophic health expenditure may suggest segments of poor people refraining from using health care because of OOP payments.

These findings reinforce the critical role that expenditure on transportation while accessing health care plays on households’ financial hardship, particularly for the poor segment and those residing in rural areas, corroborating the growing evidence particularly from Africa (McLaren *et al.*, 2014; Masiye F *et al.*, 2016; Barasa *et al.*, 2017). While not traditionally included in the OOP payment basket, this expenditure item deserves health policy attention, particularly in low- and middle-income countries with geographically difficult contexts.

The results, however, must be interpreted in light of several limitations. First, there is a high likelihood of forgone care, underestimating catastrophic health expenditure, impoverishment and financial hardship found in this analysis. Poorer households may choose not to seek care rather than face financial hardship (Xu *et al.*, 2003). Second, the BLSSs collect self-reported data on health care use and expenditures, which is prone to recall bias. Furthermore, the differing recall periods between outpatient and inpatient services, and standardizing them to monthly expenditures, may hamper the extrapolation of results. Third, the surveys are not designed to capture non-financial transfers between households that might have enabled households to meet unexpected health expenditures. Further research is necessary to monitor these findings as well as further dwell on the limitations encountered in this study.

## Conclusion

This study provides evidence on catastrophic health care expenditure and impoverishment due to OOP payments in Bhutan covering a period of 15 years. The health system needs to adapt to resolve these shortcomings, ensuring geographical access and quality of services, especially in the rural parts and regions of the country and among the less advantaged population groups. There is a need for increased investments in access to an appropriate level and quality of health services in order to enhance the effective coverage of services and reduce financial hardship. Demand-side financing and policies to reduce the burden of transport costs, especially among the poor and the vulnerable, could possibly be explored. These measures could strengthen Bhutan’s progress towards UHC.

Methodologically, building upon previous works, we used a composite measure of financial hardship combining the measures of catastrophic health expenditure and impoverishment. This may potentially ease deciphering and visualization of the financial burden of OOP payments for policymakers and researchers alike. This should contribute to prompt translation of evidence to policy and provide a comparative reference for researchers in other low- and middle-income countries.

## Supplementary Material

czac107_Supp

## Data Availability

The quantitative data underlying this article were provided by the National Statistics Bureau, Bhutan. The datasets generated during and analysed in this study will be shared on reasonable request to the corresponding author with permission of the National Statistics Bureau, Bhutan.

## Endnotes

1. 1 USD = 66.43 Ngultrum, based on the annual average for 2017 calculated by the Royal Monetary Authority of Bhutan.

## Reflexivity statement

The authors include one female and two males from two countries (Bhutan and the Netherlands) and span across multiple levels of seniority. Two of the authors specialize in health economics, while the third is a health system researcher with extensive experience in health policy and systems fieldwork in Bhutan.
