# Supplementary material for: Catastrophic health care expenditure and impoverishment in Bhutan
Source: Health Policy Plan. 2022 Dec 7;38(2):228–38. doi: 10.1093/heapol/czac107 (PMC9923371; doi:10.1093/heapol/czac107)
Supplement: czac107_Supp [file czac107_supp.zip › FINAL Appendix 1.docx]

**Appendix 1: Socio-demographic characteristics of the samples N(%)**

|  | **2007** | **2012** | **2017** |
| --- | --- | --- | --- |
| Sample size (households) | 9798 | 8968 | 11660 |
|  |  |  |  |
| Area of residence |  |  |  |
| Rural | 6856 (69.97) | 4349 (69.97) | 6714 (57.58) |
| Urban | 2942 (30.03) | 4619 (30.03) | 4946 (42.42) |
|  |  |  |  |
| Geographical region of residence |  |  |  |
| Western region | 4025 (41.08) | 4403 (49.10) | 5301 (45.46) |
| Central region | 2944 (30.05) | 2327 (25.95) | 3603 (30.90) |
| Eastern region | 2829 (28.87) | 2238 (24.96) | 2756 (23.64) |
|  |  |  |  |
| Sex of the household head |  |  |  |
| Male | 6759 (68.98) | 6560 (73.15) | 7519 (64.49) |
| Female | 3039 (31.02) | 2408 (26.85) | 4141 (35.51) |
|  |  |  |  |
| Socio-economic quintiles of households |  |  |  |
| Quintile 1 (poorest) | 1927 (19.67) | 1505 (16.78) | 2089 (17.92) |
| Quintile 2 | 1943 (19.83) | 1613 (17.99) | 2197 (18.84) |
| Quintile 3 | 1977 (20.18) | 1784 (19.89) | 2303 (19.75) |
| Quintile 4 | 2005 (20.46) | 1915 (21.35) | 2452 (21.03) |
| Quintile 5 (richest) | 1946 (19.86) | 2151 (23.99) | 2619 (22.46) |
|  |  |  |  |
| Education of household head |  |  |  |
| No formal education | 6663 (68.00) | 4691 (52.31) | 6423 (55.09) |
| Primary, secondary and vocational | 2832 (28.90) | 3576 39.88) | 4328 (37.12) |
| Bachelors and higher | 303 (3.09) | 701 (7.82) | 909 (7.80) |
|  |  |  |  |
| Employment status of household head |  |  |  |
| Employed | 6291 (64.26) | 5555 (62.03) | 9704 (83.22) |
| Unemployed | 3499 (35.74) | 3400 (37.97) | 1956 (16.78) |
| No data (missing) | 8 | 13 | 0 |
|  |  |  |  |
| Age of household head  Mean(CI) | 45.26  (44.94-45.55) | 43.69  (43.39-44.00) | 45.25  (44.99-45.52) |
| Number of household members  Mean (CI) | 5.02  (4.97-5.06) | 4.44  (4.40-4.48) | 4.17  (4.14-4.21) |
| Number of children less than 13 years of age  Mean (CI) | 1.52  (1.49-1.55) | 1.24  (1.21-1.26) | 1.07  (1.05-1.09) |
| Number of elderly (above 65) household members  Mean (CI) | 0.41  (0.40-0.42) | 0.36  (0,35-0,38) | 0.44  (0.42-0.45) |
