# Supplementary material for: Catastrophic health care expenditure and impoverishment in Bhutan
Source: Health Policy Plan. 2022 Dec 7;38(2):228–38. doi: 10.1093/heapol/czac107 (PMC9923371; doi:10.1093/heapol/czac107)
Supplement: czac107_Supp [file czac107_supp.zip › FINAL Appendix 2.docx]

**Appendix 2: Concentration curves for different measures of financial hardship**

**2017**

**2012**

**2007**
